# Clinical outcome of treatment of metastatic non-small cell lung cancer in patients harboring uncommon EGFR mutation

**DOI:** 10.1186/s12885-019-5913-9

**Published:** 2019-07-17

**Authors:** J. Chantharasamee, N. Poungvarin, P. Danchaivijitr, S. Techawatanawanna

**Affiliations:** 1 Division of Medical Oncology, Department of Medicine, Faculty of Medicine Siriraj Hospital, Mahidol University, Bangkok, Thailand; 2 Clinical Molecular Pathology Laboratory, Department of Clinical Pathology, Faculty of Medicine Siriraj Hospital, Mahidol University, Bangkok, Thailand

**Keywords:** Prevalence, Characteristics, Clinical outcomes, Metastatic non-small cell lung cancer (NSCLC), Epidermal growth factor receptor (EGFR) mutation, Siriraj hospital

## Abstract

**Background:**

Uncommon epidermal growth factor receptor (EGFR)-mutant non-small cell lung cancer (NSCLC) is a rare subset of NSCLC. The aim of this study was to investigate the prevalence, characteristics, and clinical outcomes of metastatic NSCLC harboring uncommon EGFR mutation at Thailand’s largest national tertiary hospital. The secondary objective was to compare treatment efficacy between EGFR-tyrosine kinase inhibitor (EGFR-TKI) and chemotherapy.

**Methods:**

This retrospective chart review included patients that were tested for EGFR-mutation NSCLC during 2014–2018. Patient demographic and clinical data, treatment data, and outcome data were collected and analyzed.

**Results:**

Of the 681 patients that were evaluated for EGFR mutation, 317 (47.0%) had EGFR-mutant NSCLC, and 28 (8.8%) of those harbored uncommon EGFR mutations. The median follow-up was 19.1 months. History of tobacco use was reported in 50% of patients. The most common single mutation among uncommon EGFR was exon 20 insertion (*n* = 6), followed by L861Q (*n* = 5) and G719X (*n* = 4). Thirteen (46%) patients had compound mutations. One hundred percent of male patients with G719X mutation were smokers. Sixteen of 28 patients were treated with EGFR-TKI. Most received first-generation EGFR-TKI, and 29% were treated with chemotherapy alone. The objective response rate was 37.5% in the EGFR-TKI group. Median progression-free survival (PFS) in the EGFR-TKI group was 10.2 months. Median PFS among the 8 patients in the chemotherapy group that received first-line platinum doublet was 6.5 months. Three-year overall survival (OS) among 28 patients was 34%. Three-year OS was significantly better in patients treated with EGFR-TKI.

**Conclusions:**

Uncommon EGFR mutations was detected in 8.8% of EGFR-mutant NSCLC. Exon 20 insertion was the most common mutation, and 50% of patients had history of tobacco use. First- or second-generation EGFR-TKI demonstrated greater OS benefit than platinum-doublet chemotherapy among patients harboring uncommon EGFR-mutant NSCLC. Survival outcomes were comparable to those reported from previous large cohort studies.

## Background

Non-small cell lung cancer (NSCLC) is the most common cancer, and is the second most common cause of cancer-related death worldwide [1]. Studies in Asian population revealed a prevalence of EGFR mutation of 40–60%, which is higher than the 10–30% reported in Caucasian population [2, 3]. Standard systemic treatment for patients with stage IIB/IV NSCLC with sensitizing epidermal growth factor receptor (EGFR) mutation includes either first-generation EGFR-tyrosine kinase inhibitor (TKI) (gefitinib or erlotinib) or second-generation TKI (afatinib). Third-generation TKI (osimertinib) is the most recently approved agent, but it is not yet widely available in Thailand. Previous phase III studies reported a response rate by first- or second-generation EGFR-TKI of 50–60%, with significantly longer progression-free survival (PFS) than platinum-doublet chemotherapy (CMT) [4–9]. The majority of patients in those large cohort studies had common (sensitizing) EGFR mutation, with either deletion of exon19 (del19) or point mutation Leu858Arg. In patients harboring uncommon EGFR mutation, including singlet uncommon or doublet/multiple (complex) mutations, the prevalence of different types of mutations and the characteristics of patients varied among cohorts and geographic data range from 5 to 20% [3, 10–13]. The benefit of EGFR-TKI treatment in patients with metastatic NSCLC that harbor uncommon EGFR mutation is still being investigated and debated [14, 15]. A study in Japanese population found inferior overall survival in patients treated with gefitinib compared to those treated with CMT [4]. In contrast, studies from China showed comparable or superior survival outcome by first-generation EGFR-TKI compared to CMT [15, 16]. The most recent data from combined post-hoc analysis of the LUX-Lung 2, 3, and 6 trials revealed lower median overall survival (OS) in patients treated with afatinib (19.4 months) compared to those treated with platinum-doublet CMT (30.2 months) among patients with uncommon or complex mutation [12].

Improved understanding of patients with metastatic NSCLC that harbor uncommon EGFR mutation may improve patient diagnosis, treatment, and outcomes. Accordingly, the aim of this study was to investigate the prevalence, characteristics, and clinical outcomes of metastatic NSCLC harboring uncommon EGFR mutation at Siriraj Hospital – Thailand’s largest national tertiary referral center. The secondary objective was to compare treatment efficacy in patients with this condition between EGFR-tyrosine kinase inhibitor (EGFR-TKI) and chemotherapy.

## Methods

### Study design and patient selection

This retrospective study included patients diagnosed with stage IIIB-IV non-small cell lung cancer who were tested for EGFR-mutation at the Faculty of Medicine Siriraj Hospital, Mahidol University, Bangkok, Thailand during the 1 January 2014 to 31 December 2018 study period. All included patients received one of the following five treatments: gefitinib, erlotinib, afatinib, chemotherapy or best supportive care. Patients having one or more of the following were excluded: no follow-up data, no post-treatment imaging, and/or received tyrosine kinase inhibitor prior to molecular testing. The following patient demographic, clinical, and molecular characteristics were collected: age, gender, smoking status, histology, specimen type, mutation type, type of EGFR-TKI, line of TKI treatment, line of chemotherapy treatment, and subsequent targeted therapy. Progression-free survival (PFS) was defined as the interval from the first day of treatment by EGFR-TKI in the TKI group, and the first date of treatment by first-line platinum doublet chemotherapy in the CMT group until progression of disease or date of death from any cause (whichever occurred first). Overall survival was defined as the interval between the date of diagnosis of stage IIIB/IV (incurable) NSCLC and the date of death from any cause.

### EGFR mutation testing

All mutation testing of specimens was performed at the Clinical Molecular Pathology Laboratory, Department of Clinical Pathology, Faculty of Medicine Siriraj Hospital, Mahidol University. Analysis of EGFR mutation status in tissue and plasma samples was performed using cobas EGFR Mutation Test (F. Hoffmann-La Roche, Switzerland) or validated in-house allele specific PCR assays (reference: http://www.ncbi.nlm.nih.gov/pubmed/24370549).

### Statistical analysis

Data analysis was performed using SPSS Statistics version 21 (SPSS, Inc., Chicago, IL, USA). Patient characteristics and treatment outcomes are described using descriptive statistics. Categorical variables are reported as frequency and percentage. Continuous variables are reported as mean ± standard deviation for normally distributed variables, and as median and range (minimum and maximum) for non-normally distributed data. Kaplan-Meier survival analysis was used to estimate overall survival (OS) and progression-free survival (PFS), with comparison between groups by log-rank test. In order to identify variables independently associated with OS and PFS, variables with a *p*-value< 0.05 in univariate analysis were included in multivariate analysis by Cox proportional hazard regression. A two-tailed *p*-value less than 0.05 was considered statistically significant for all tests. The 4 patients who received best supportive care only were excluded from progression-free and overall survival analysis.

## Results

### Baseline characteristics

Of the 681 patients that were evaluated for EGFR mutation, 317 (47.0%) had EGFR-mutant NSCLC, and 28 (8.8%) of those harbored uncommon EGFR mutations. Eighteen (64%) of 28 patients were male. The median age at diagnosis was 67 (range: 53–80) years. Twenty-four of 28 (85.7%) were stage 4 at the first diagnosis. History of tobacco use was found in 50% of patients. The majority of histology was adenocarcinoma, with only one specimen found to be squamous cell carcinoma. ECOG 0–1 was equal between TKI and chemotherapy group (15 of 16 and 7 of 8, respectively). 64% presented with extra-pulmonary metastasis, and 17% had a CNS metastasis. Five of 28 specimens were plasma only. Fifteen patients had single mutation, and 13 patients had complex or compound mutation. The most common single mutation was exon20 insertion (Ex20Ins) (*n* = 6), followed by Leu861Gln (L861Q) (*n* = 5) and Gly719Xaa (G719X) (*n* = 4). Of the 13 patients with compound mutations, 4 had G719X plus Ser768Ile (S768I), 4 had de novo T790 M plus either Leu858Arg (L858R) or deletion(del)19, 2 had L858R plus del19, 1 had L858R plus Ex20Ins, 1 had del19 plus KRAS mutation in treatment-naïve, and 1 with Gly719Xaa plus E709A was found in squamous cell carcinoma. None of S768I mutation specimens were singlet (Table 1). According to the mutation subtype, Ex20Ins mutation had a higher female to male ratio compare to others, and all of these patients had an extra-pulmonary metastasis. 100% of male patients with G719X mutation were smokers (Table 2).

**Table 1:** Baseline characteristics and pathology of patients with uncommon EGFR mutations

Characteristics	Total (*n* = 28)	EGFR-TKI (*n* = 16)	Chemotherapy alone (*n* = 8)	Best supportive care (*n* = 4)
Age (years), median (range)	68 (53–80)	67.5 (53–80)	67.5 (54–75)	75 (68–79)
Gender, n (%)				
Female	10 (35.7)	4 (14.2)	4 (14.2)	2 (7.1)
Male	18 (64.2)	12 (42.8)	4 (14.2)	2 (7.1)
ECOG				
- 0–1	23 (82.1)	15 (53.5)	7 (25.0)	1 (3.5)
- 2	2 (7.1)	0 (0)	1 (3.5)	1 (3.5)
- 3	3 (10.7)	1 (3.5)	0 (0)	2 (7.1)
Stage at diagnosis				
I	1 (3.5)	0 (0)	1	0 (0)
II	0 (3.5)	0 (0)	0 (0)	0 (0)
IIIA	1 (3.5)	0(0)	0 (0)	1 (3.5)
IIIB	2 (7.1)	2 (7.1)	0 (0)	0 (0)
IV	24 (85.7)	14 (50)	7 (25)	3 (10.7)
Specimen/site biopsy, n (%)				
Lung parenchyma	11 (39.2)	7 (25)	3 (10.7)	1 (3.5)
Pleural nodule	6 (21.4)	3 (10.7)	2 (7.1)	1 (3.5)
Lymph node	1 (3.5)	1 (3.5)	0 (0)	0 (0)
Bone	1 (3.5)	1 (3.5)	0 (0)	0 (0)
Other	1 (3.5)	1 (3.5)	0 (0)	0 (0)
Cytology	3 (10.7)	2 (7.1)	1 (3.5)	0 (0)
Plasma only	5 (17.8)	2 (7.1)	2 (7.1)	1 (3.5)
Smoking status, n (%)				
Never smoked	14 (50)	8 (28.5)	4 (14.2)	2 (7.1)
Ex-smoker/smoker	14 (50)	8 (28.5)	4 (14.2)	2 (7.1)
Histology, n (%)				
Adenocarcinoma	27 (96.4)	15 (53.5)	8 (28.5)	4 (14.2)
Squamous cell	1 (3.5)	1 (3.5)	0 (0)	0 (0)
Extra- pulmonary metastasis, n (%)	18 (64.2)	10 (35.7)	7 (25)	1 (3.5)
Present of CNS metastasis, n (%)	5 (17.8)	1 (3.5)	4 (14.2)	0 (0)
Mutation subtypes, n (%)				
Single mutation				
Exon 20 insertion	6 (21.4)	0 (0)	5 (17.8)	1 (3.5)
Exon 21 L861Q	5 (17)	4 (14.2)	1 (3.5)	0 (0)
Exon 18 G719X	4 (14.2)	3 (10.7)	0 (0)	1 (3.5)
Compound mutation				
G719X+ Exon 20 S768I	4 (14.2)	2 (7.1)	0 (0)	2 (7.1)
De novo T790 M + L858R	1 (3.5)	0 (0)	1 (3.5)	0 (0)
De novo T790 M + del19	3 (10.7)	3 (10.7)	0 (0)	0 (0)
L858R + del19	2 (7.1)	2 (7.1)	0 (0)	0 (0)
L858R + Ex20Ins	1 (3.5)	0 (0)	1 (3.5)	0 (0)
Del19 + KRAS	1 (3.5)	1 (3.5)	0 (0)	0 (0)
G719X + E709A	1 (3.5)	1 (3.5)	0 (0)	0 (0)
EGFR-TKI, n (%)				
Erlotinib	8 (28.5)			
Gefitinib	6 (21.4)			
Afatinib	2 (7.1)			
Line of TKI treatment, n (%)				
First-line	7 (25)			
Second-line	7 (25)			
Third-line or later	2 (7.1)			
Subsequent osimertinib, n (%)				
Del19 + L858R with acquire T790 M	1 (3.5)			
De novo T790 M + del19	2 (7.1)

**Table 2:** Clinical characteristics by EGFR mutation subtypes

	Single mutation			Compound mutation		
	G719X (*n* = 4)	L861Q (*n* = 5)	Exon 20 Ins (*n* = 6)	Combine with uncommon mutation (*n* = 7)^a^	Del19 plus L858R (*n* = 2)	Combine with de novo T790 M (*n* = 4)
Median age (years)	72.5	69	68.5	68	59 and 80	65
Sex						
Female	1	1	4	2	0	2
Male	3	4	2	5	2	2
Smoking history						
Yes	3	3	2	4	1	1
No	1	2	4	3	1	3
Extra-pulmonary metastasis						
Yes	2	2	6	1	1	4
No	2	3	0	3	1	0
Present of CNS metastasis						
Yes	1	1	0	1	0	2
No	3	4	6	6	2	2

^a^4 of G719X plus S768I, 1 of G719X plus E709A, 1 of L858R plus Exon20Ins and 1 of del19 plus KRAS mutant

### Treatment outcome

**Fig. 1:**
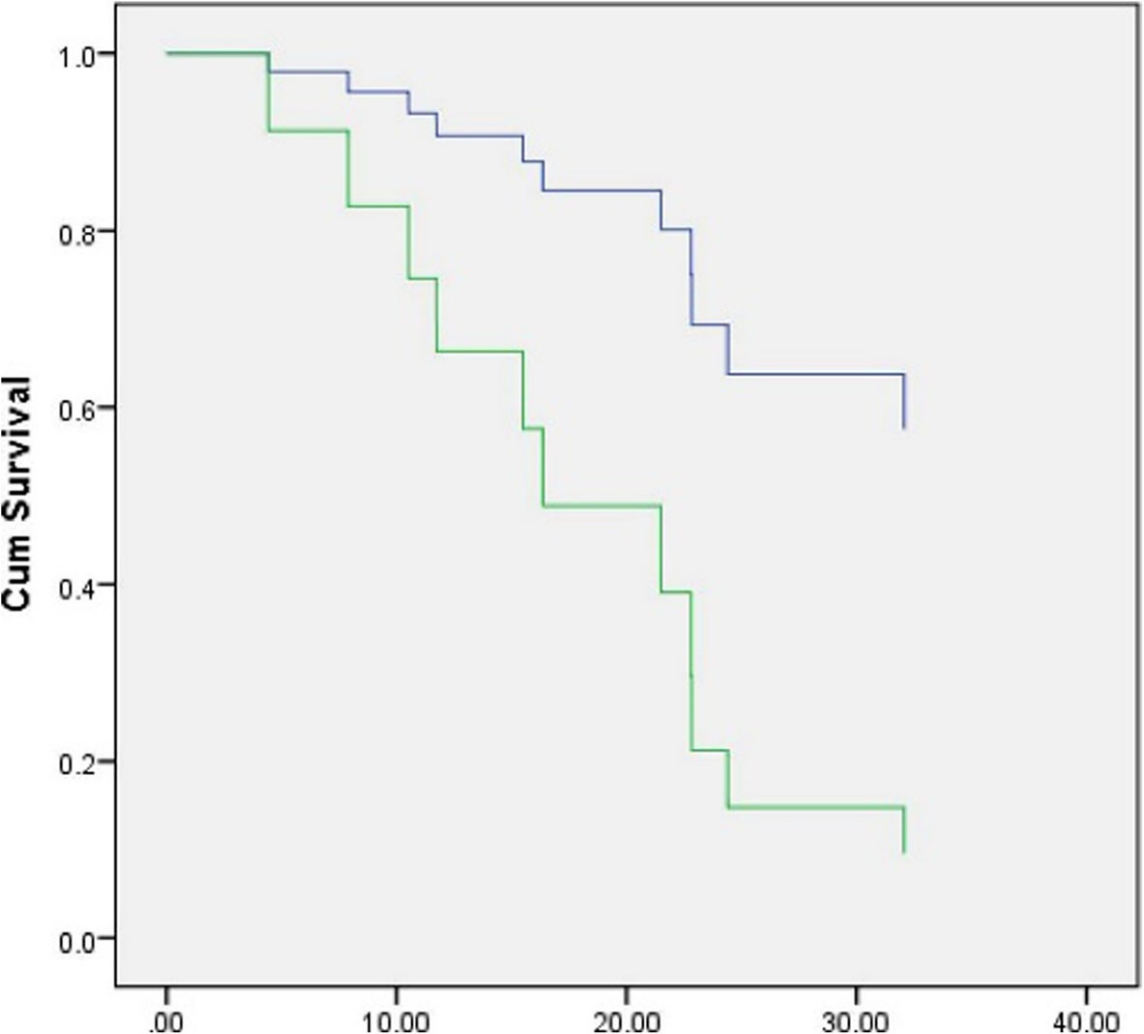
Overall survival by treatment (blue line = TKI-treated group, green line = CMT-treated group)

**Fig. 2:**
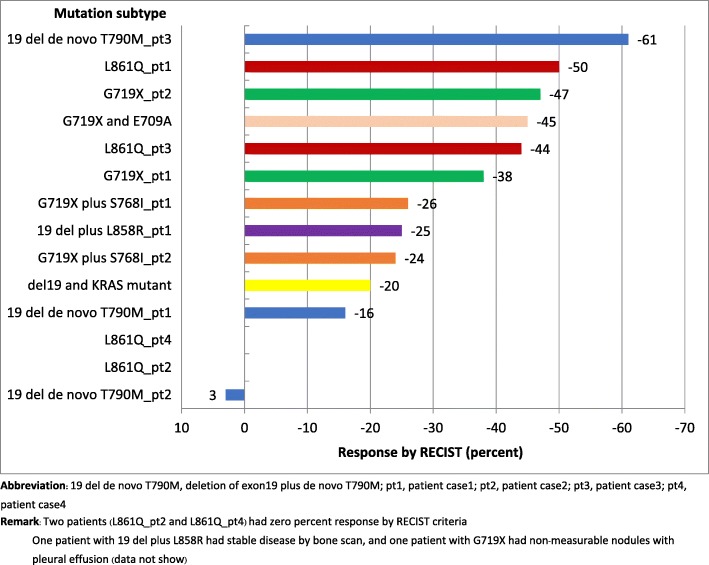
Best response to EGFR-TKI by RECIST criteria according to mutation subtype (*n* = 14). Abbreviation: *19 del de novo T790M* deletion of exon19 plus de novo T790M, *pt1* patient case1, *pt2* patient case2, *pt3* patient case3, *pt4* patient case4. Remark: Two patients (L861Q_pt2 and L861Q_pt4) had zero percent response by RECIST criteria One patient with 19 del plus L858R had stable disease by bone scan, and one patient with G719X had non-measurable nodules with pleural effusion (data not show)

The median follow-up was 19.1 months. Sixteen of 28 (57%) patients were treated with EGFR-TKI, as follows: 8 with erlotinib, 6 with gefinitib, and 2 with afatinib. No patients with Ins20 mutation received EGFR-TKI. Seven patients received TKI as first-line treatment, and 7 and 2 patients received TKI as second- and third-line treatment, respectively. Three of four patients with de novo T790 M received EGFR-TKI (gefitinib or erlotinib). Eight patients (29%) received platinum doublet chemotherapy without TKI exposure, and 4 patients received best supportive care. Median overall *survival (*OS) in all patients was 18.9 months (range: 0.37–73.6). Median OS in the TKI and CMT groups was 23.6 and 15.9 months, respectively. The 3-year OS rate was significantly higher in the TKI group than in the CMT group (53% vs. 17%, respectively; *p* = 0.014 by log-rank test) (Fig. 1). Median progression-free survival (mPFS) in the TKI group was 10.2 months. The PFS was 7.8 months in patients who received TKI as a first-line treatment. Regarding PFS by mutation subtype, the longest PFS (22.5 months) was observed in a patient that harbored G719X plus S768I that was treated with erlotinib as third-line therapy. In the 3 patients with de novo T790 M mutation combined with deletion19 that received TKI, the PFS was 6.5, 9.7, and 11.8 months, respectively. Two of those 3 patients received subsequent osimertinib, and their OS was 22.8 and 73.6 months, respectively. The patient with del19 plus L858R had acquired T790 M mutation that received subsequent osimertinib had an OS of 67.8 months. One patient with squamous cell carcinoma that harbored G719X plus E709A had PFS of 17.6 months (Table 3). No significant difference in mPFS was observed between single and compound mutation in whom treated with TKI using Cox proportional hazard regression (Table 4). The objective response rate (ORR) defined as complete or partial response (CR/PR) by RECIST criteria was 37.5% (6 of 16 patients) and the clinical benefit rate was 68.7% (11 of 16 patients) in the TKI group (Fig. 2). The mPFS among the 8 patients that received first-line platinum doublet in the chemotherapy group was 6.5 months.

**Table 3:** Treatment outcome according to mutation subtype in patients treated with EGFR-TKI

Mutation subtype	PFS range (months)*
De novo T790 M plus del19 (*n* = 3)	6.5–11.8
L861Q (*n* = 4)	1.2–12.6
G719X (*n* = 3)	0.5–2.8
G719X plus S768I (*n* = 2)	7.8–22.5
G719X plus E709A (*n* = 1)	17.6
Del19 plus L858R (*n* = 2)	13.3–16.4
Del19 plus KRAS (*n* = 1)	11.9

*Abbreviations*: *PFS* progression-free survival

*According to RECIST (response evaluation criteria in solid tumors) criteria

**Table 4:** Univariate analysis for PFS after EGFR-TKI treatment

Variable	95% CI	*p* value
Age: ≤ 65 vs. > 65 years	0.10–1.74	0.23
Sex: male vs. female	0.45–30.31	0.21
History of smoking: yes vs. no	0.12–1.99	0.32
Mutation: single vs. compound	0.13–3.82	0.70
Line of TKI treatment: first vs. later line	0.33–4.70	0.74

### Correlation analysis

Univariate and multivariate analysis using Cox proportional hazard regression was performed to identify factors, including gender, smoking status, mutation subtype, and line of TKI therapy, that are independently associated with survival outcome in patients that received TKI therapy. No significant difference or association was observed. Multivariate analysis was not performed due to no significant difference in univariate analysis (Table 4).

## Discussion

EGFR tyrosine kinase inhibitors are globally established as a first-line treatment for advanced non-small cell lung cancer patients with a sensitizing EGFR mutation. Mutations of exon 21 Leu858Arg and exon 19 deletion are generally sensitive to all generations of EGFR-TKI, but the effect and benefit of EGFR-TKI in NSCLC harboring uncommon or compound EGFR mutations is less clear. The low prevalence and heterogeneity of mutational subtypes limits the ability of clinical trials to develop paradigms for standard treatment. Previous studies reported response rates by first-generation EGFR-TKI that ranged from 23.3–66.6% [13, 17–20]. The aim of this study was to investigate the prevalence, characteristics, and clinical outcomes of metastatic NSCLC harboring uncommon EGFR mutation, to compare treatment efficacy in patients with this condition between EGFR-tyrosine kinase inhibitor (EGFR-TKI) and chemotherapy. This study revealed a prevalence of EGFR mutation of 47%, which was comparable to the rates reported from previous studies [2]. However, the prevalence of uncommon or combined mutation was 8.8%, which is lower than the 13.9% that was reported from a large Chinese study [12, 16]. The prevalence of uncommon or combined mutation in North America and Europe was reported to range from 5 to 20% [8, 21]. The most frequent single uncommon mutation in this study was exon20 insertion (21%; 6 of 28), followed by L861Q (18%; 5 of 28), which is consistent with the percentages reported in the LUX-Lung 3 and 6 studies [12]. The median progression-free survival in our TKI cohort was 10.2 months, which is similar to the TKI group of Asian population in the Lux-Lung 3 and Lux-Lung 6 joint study, but slightly longer than the rates reported from most Caucasian studies [12, 20, 21]. The objective response rate of 37.5% and the clinical benefit rate of 68.7% was comparable to the previous studies [12, 13, 16].

In patients with L861Q mutation, The previous studies reported a median PFS ranging from 1.9–8.2 months [12, 16], which comparable to 11.7 months of our study.

In the present study, patients with mutation at S768I, which was reported to be a potential EGFR-TKI sensitizing NSCLC, had PFS that ranged from 7.8 to 22.5 months, which is consistent with the PFS ranges observed in previous studies [16, 22]. None of the S768I mutation cases had single mutation, and they coexisted with G719X in every case, which is similar to previous reports [11, 22, 23]. We observed a PFS range of 0.5–2.8 in patients with single G719X mutation, which is shorter than previously published ranges [16, 23, 24]. The PFS range in patients harboring doublet G719X mutation plus others was 7.8–22.5 months, which is longer than the range found in a large study by Shi, et al. (range: 1–8.6 months) [16].

We also observed in our study a patient with a very rare doublet mutation of G719X plus E709A within a squamous cell carcinoma specimen obtained by core needle biopsy, and that patient had PFS of 17.6 months. In vitro evidence suggests that compound E709A reduced the efficacy of TKI when compared to G719X alone [25–27]. However, Jenn Y, et al. reported 2 cases of adenocarcinoma with G719C plus E709A mutation that responded to first-generation EGFR-TKI, with PFS 7.3 and 14.9 months, respectively [28]. Combined G719 plus E709 mutation within squamous cell specimen has not been previously reported.

Del19 plus KRAS mutation was also found in one patient in the present study. This combination mutation is thought to be exclusive to EGFR-mutation NSCLC and it is generally resistant to EGFR-TKI, with a prevalence of less than 1% [3, 29–32]. However, our patient had partial response for a PFS of 11.9 months, which is consistent with previous case series that reported durable disease control ranging from 9 to 29 months by gefitinib and erlotinib [17, 33, 34]. The percentage of KRAS-mutant clones within the tumor, and the type of variant of KRAS-mutant codon were proposed to be factors that impact heterogeneous outcome, but no clear association could be established [33–36].

The impact of de novo T790 M on the responsiveness of first- and second-generation EGFR-TKI has been widely established. Pre-treated T790 M-positive NSCLC was associated with decreased PFS compared to NSCLC without T790 M [37, 38]. Our study showed 3 patients with PFS of 6.5, 9.7, and 11.8 months, respectively, by first-generation EGFR-TKI. The variation in PFS among patients with de novo T790 M was also reported in EURTAC subanalysis [38].

The insertion 20 mutation was reported to be an EGFR-TKI-resistant mutation in the previous study [39, 40]. None of our patient was treated with TKI.

Most studies of uncommon mutation reported only rare subtype or combine rare subtype with sensitizing mutation. In our study, we also found doublet common mutation of deletion 19 plus L858R. Two patients with these mutations in our study had a slightly longer DFS (13.3 and 16.4 months) than in single mutation either del19 or L858R EGFR mutant NSCLC, which reported in the previous phase 3 studies (9.5–13.6 months) [5, 7, 12, 41].

Regarding patients in the chemotherapy group, the median progression-free survival in first-line platinum doublet treatment was 6.5 months, which is comparable with previous reports [7, 12, 16]. For overall survival, the median OS of all 28 uncommon mutation patients was 18.9 months. By treatment group, the median OS in the TKI group and the CMT group was 23.6 months and 15.9 months, respectively, which is comparable with the rates reported from previous large-scale studies [3, 12, 13, 16].

We used univariate and multivariate analyses to identify factors independently associated with survival outcome. Previous study revealed that doublet or multiple EGFR mutation associated with similar or poorer PFS by TKI treatment compared to singlet uncommon mutation [16, 18, 23, 24, 42]. We included factors like age, line of TKI treatment, and mutation subtype in our analysis, but none of these factors significantly associated with PFS outcome.

### Limitations

This study has some mentionable limitations. First, the retrospective nature of this study makes it vulnerable to incomplete or missing data. Second, the uncontrolled line of treatment between TKI and CMT permitted us to report only PFS for the TKI group compared to the PFS results reported from previous studies, but not compared to the PFS results from our CMT group. We, therefore, were unable to determine if there was any benefit of TKI over CMT relative to PFS. Third, we could not evaluate the impact of a number of subsequent treatment lines in each arm of patients due to incomplete follow-up data outside our hospital after referral. Forth and last, three of the patients that we included that had either de novo or acquired T790 M (2 patients) or acquired T790 M in doublet mutation del19 plus L858R (1 patient) were treated with subsequent osimertinib. The OS of these 3 patients ranged from 22.8–73.6 months, and this could have skewed the OS rate in the TKI group.

## Conclusion

Uncommon EGFR mutations was detected in 8.8% of EGFR-mutant NSCLC. Exon 20 insertion was the most common mutation, and 50% of patients had history of tobacco use. First- or second-generation EGFR-TKI demonstrated greater OS benefit than platinum-doublet chemotherapy among patients harboring uncommon EGFR-mutant NSCLC. Survival outcomes were comparable to those reported from previous large cohort studies.

## Data Availability

The datasets used and/or analysed during the current study are available from the corresponding author on reasonable request.

## Abbreviations

CMT: Chemotherapy
del19: deletion of exon19
EGFR: Epidermal growth factor receptor
G719X: Gly719Xaa
L861Q: Leu861Gln
NSCLC: Non-small cell lung cancer
OS: Overall survival
PFS: Progression-free survival
S768I: Ser768Ile
TKI: Tyrosine kinase inhibitor
